# Development of a dedicated 3D printed myocardial perfusion phantom: proof-of-concept in dynamic SPECT

**DOI:** 10.1007/s11517-021-02490-z

**Published:** 2022-01-19

**Authors:** Marije E. Kamphuis, Gijs J. de Vries, Henny Kuipers, Marloes Saaltink, Jacqueline Verschoor, Marcel J. W. Greuter, Riemer H. J. A. Slart, Cornelis H. Slump

**Affiliations:** 1 Multi-Modality Medical Imaging (M3i) Group, Faculty of Science and Technology, Technical Medical Centre 2386, University of Twente, P.O. Box 217, 7500 AE Enschede, The Netherlands; 2 Robotics and Mechatronics (RaM) Group, Faculty of Electrical Engineering Mathematics and Computer Science, Technical Medical Centre, University of Twente, Enschede, The Netherlands; 3 Department of Nuclear Medicine, Ziekenhuis Groep Twente, Hengelo, The Netherlands; 4 Medical Imaging Centre, Department of Nuclear Medicine and Molecular Imaging, University Medical Center Groningen, University of Groningen, Groningen, The Netherlands; 5 Biomedical Photonic Imaging Group, Faculty of Science and Technology, Technical Medical Centre, University of Twente, Enschede, The Netherlands

**Keywords:** Perfusion/flow phantom, 3D printing, Myocardial perfusion imaging, Reference experiments

## Abstract

We aim to facilitate phantom-based (ground truth) evaluation of dynamic, quantitative myocardial perfusion imaging (MPI) applications. Current MPI phantoms are static representations or lack clinical hard- and software evaluation capabilities. This proof-of-concept study demonstrates the design, realisation and testing of a dedicated cardiac flow phantom. The 3D printed phantom mimics flow through a left ventricular cavity (LVC) and three myocardial segments. In the accompanying fluid circuit, tap water is pumped through the LVC and thereafter partially directed to the segments using adjustable resistances. Regulation hereof mimics perfusion deficit, whereby flow sensors serve as reference standard. Seven phantom measurements were performed while varying injected activity of ^99m^Tc-tetrofosmin (330–550 MBq), cardiac output (1.5–3.0 L/min) and myocardial segmental flows (50–150 mL/min). Image data from dynamic single photon emission computed tomography was analysed with clinical software. Derived time activity curves were reproducible, showing logical trends regarding selected input variables. A promising correlation was found between software computed myocardial flows and its reference ($\rho$= − 0.98; *p* = 0.003). This proof-of-concept paper demonstrates we have successfully measured first-pass LV flow and myocardial perfusion in SPECT-MPI using a novel, dedicated, myocardial perfusion phantom.

**Graphical abstract:**

This proof-of-concept study focuses on the development of a novel, dedicated myocardial perfusion phantom, ultimately aiming to contribute to the evaluation of quantitative myocardial perfusion imaging applications.

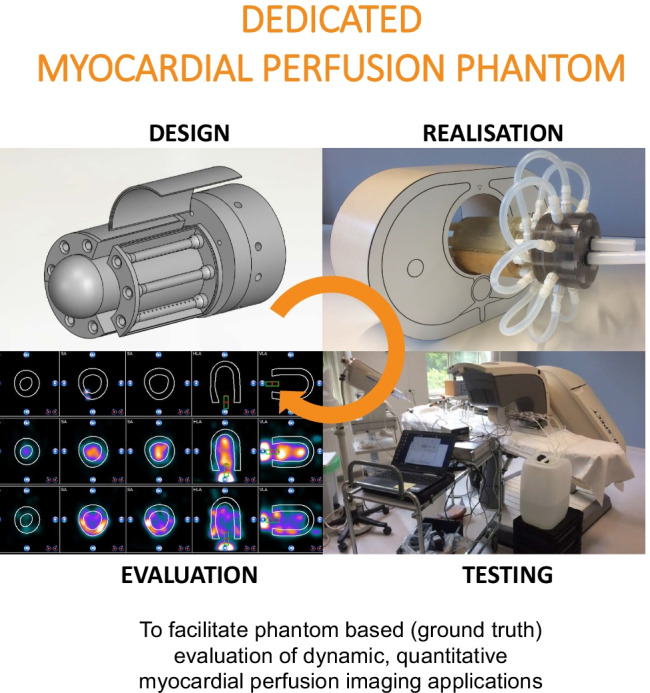

**Supplementary Information:**

The online version contains supplementary material available at 10.1007/s11517-021-02490-z.

## Introduction

Absolute quantification in dynamic rest and stress myocardial perfusion imaging (MPI) is becoming more routine in the assessment of myocardial ischemia and the diagnosis of coronary artery disease [1–3]. Patients suffering from balanced ischemia, complicated previous multiple coronary interventions and microvascular dysfunction may especially benefit from measurement of myocardial blood flow (MBF) and flow reserve (MFR) [4, 5]. This is in addition to visual evaluation or semi-quantitative approaches. Apart from a higher diagnostic accuracy in these particular patient groups, absolute MPI might also facilitate standardised assessment, with the aim of implementing universal cut-off values of flow estimates, e.g. in revascularisation decision-making [6].

In previous decades, many studies have focused on quantitative MPI with positron emission tomography (PET) [5–7]. Currently, quantitative MPI expands to other imaging domains, including computed tomography (CT) [8, 9], magnetic resonance imaging (MRI) [10], single photon emission CT (SPECT) [11–13] and ultrasound (US) [14]. Validation of MBF and MFR quantification, and underlying variety in blood flow models [15], is important in order to achieve adequate, safe and widespread clinical implementation and interpretation. Moreover, if one indicates and appreciates the possibilities of specific hard- and software, one might learn how to deal with current limitations. Perfusion phantom studies can contribute to this unmet need for robust quality assessment due to the controlled setup and use of flow sensors as reference standard.

Our aim is to contribute to the evaluation of multimodal dynamic MPI applications using perfusion phantom models. Based on a previous literature search [16], current available myocardial perfusion phantoms are predominantly static representations (no flow component) or lack evaluation capabilities regarding clinical software [16,17,18]. This proof-of-concept paper describes the design and realisation of a dedicated 3D printed myocardial perfusion phantom and initial performance testing in dynamic SPECT-MPI.

## Materials

### The myocardial perfusion phantom

**Fig. 1:**
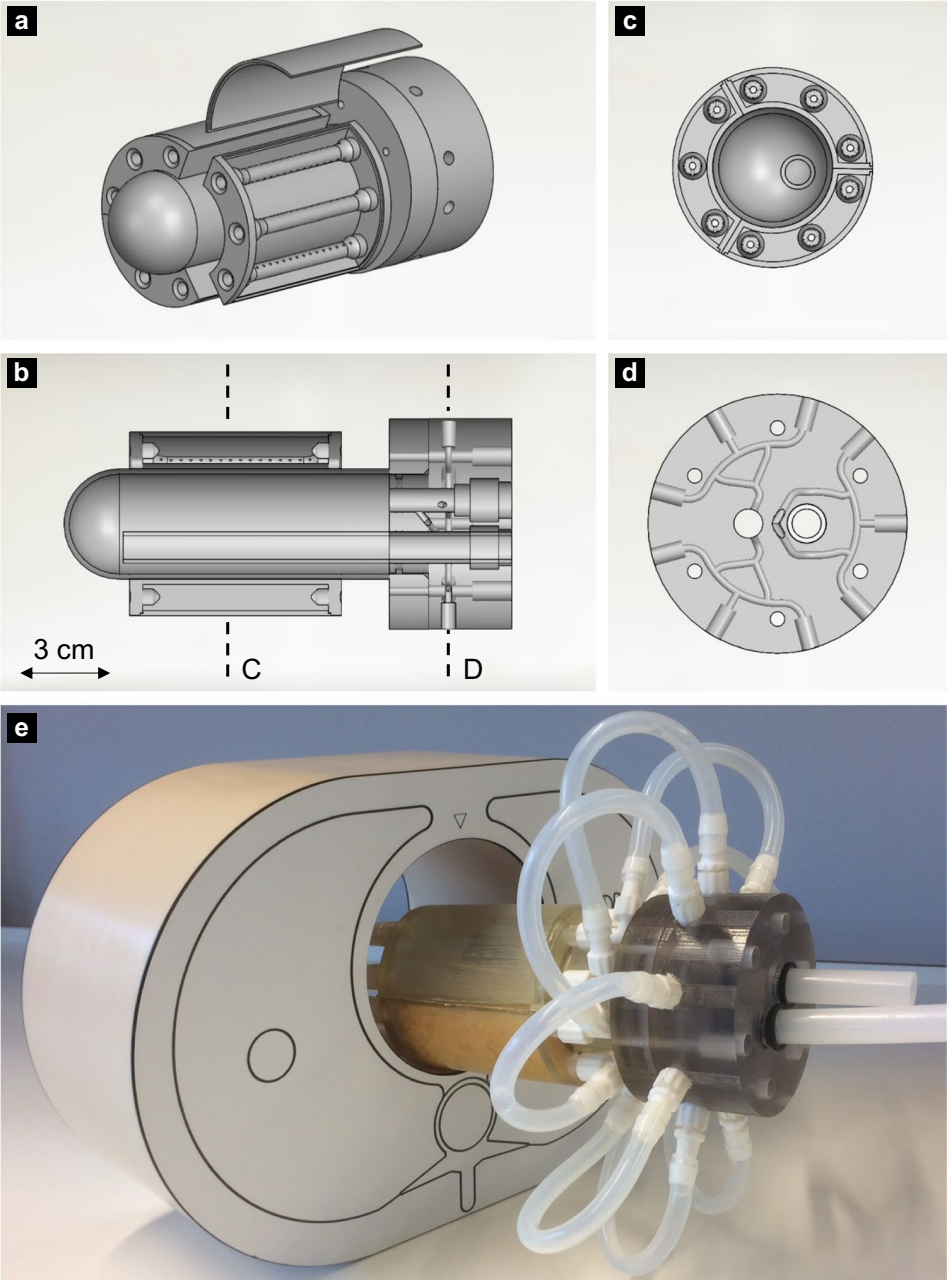
(Schematic) representation of the 3D printed myocardial perfusion phantom. (**a**) The modular phantom design including left ventricular base unit and three add-on myocardial segments. These segments can consist of different inlays. In this example, a second compartment is simulated using three perforated tubes. (**b**) a longitudinal section of the phantom in which dotted lines C and D visualise the cross-sections as depicted in respectively (**c**) and (**d**). (**e**) The overall cardiac phantom, including connectors and tubing, inserted into a thorax phantom

The myocardial perfusion phantom has the shape and size of a normal to hypertrophic male LV at end-diastolic phase. The phantom consists of a modular stationary setup, including a LV base unit and three identical add-on myocardial segments (Fig. 1). All components are designed for 3D printing (Objet260 Connex3, Stratasys, Israel) enabling rapid prototyping.

The base unit contains a connector unit and a LV cavity. To prevent printing of enclosed volumes, the parts are printed separately and assembled afterwards. As can be seen in Fig. 1(B), the main tube enters the overall cavity at the right-hand side and opens just before the apex (left-hand side) to direct the in- and outgoing flow. The LV cavity is a cylinder that is spherically shaped at the apex. The outgoing fluid within the connector unit flows into the aorta and branches into three myocardial segments (Fig. 1(C, D)).

The three myocardial segments correspond to the three main coronary territories, i.e. regions supplied by the left anterior descending coronary artery (LAD), right coronary artery (RCA) and the left circumflex coronary artery (LCX). Simulation of the apex was disregarded to facilitate comparability. The myocardial segment inlay can alter per measurement due to its modular add-on design (see Phantom Measurements). The phantom fits in an anthropomorphic thorax phantom (QRM GmbH, Moehrendorf, Germany) to obtain adequate X-ray attenuation profiles. Figure 1(E) shows the 3D printed myocardial perfusion phantom including connectors and tubing.

### The fluid circuit

**Fig. 2:**
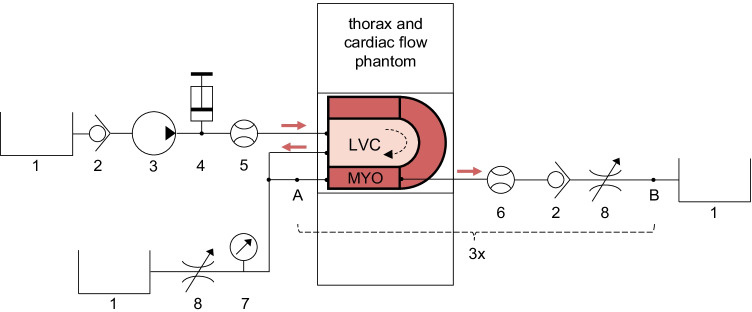
Fluid circuit diagram of measurement set-up. The phantom simulates first-pass left ventricular flow and myocardial perfusion. The cardiac phantom is inserted into a thorax phantom, comprising the scanner’s field of view. The myocardium (MYO) consists of three segments surrounding the left ventricular cavity (LVC). The individual components include: 1) water reservoirs, 2) one-way check valves, 3) an immersion pump, 4) a clinical contrast media injector, 5) an ultrasonic flow sensor, 6) turbine flow sensors, 7) a pressure sensor, and 8) adjustable resistances. The components between A and B concern one myocardial segment circuit. In total there are three such circuits connected in parallel. Water flows from top left to bottom left and right.

The measurement setup generates first-pass flow through the LVC and downstream myocardial segments (Fig. 2). The thorax phantom, containing the cardiac perfusion phantom, covers the scanner’s field of view. Tap water is pumped from a reservoir towards the LVC. Before the water enters the LVC, a radiotracer bolus can be administered to the stream using a clinical contrast media injector. After the injection, the radiotracer bolus flows through the LVC and is increasingly mixed and diluted. The outgoing tube represents the aorta and branches into three parallel-connected coronary arteries. These arteries split further into nine coronary branches of similar length that connect the three surrounding myocardial segments. Each segment ends in three coronary veins that merge into one. The diluted radiotracer concentration, passing through the aorta and three myocardial segments, is collected in two separate reservoirs. Simulation of radiotracer recirculation falls outside the scope of this study. Two sizes of plastic and silicone tubing are used (Ø_inner_ = 10 and 5 mm) to differentiate between the aorta and coronary arteries/veins.

Flow sensors measure the flow through the left ventricle (UF08B, ultrasonic flowmeter, Cynergy 3, UK) and in each myocardial segment (FCH-m-POM-LC, low flow turbine flowmeter, B.I.O-TECH, Germany). Four adjustable resistances are placed in the fluid circuit to set the flow ratio between aorta and individual myocardial segments. In this way, it is also possible to simulate global and regional perfusion deficits. An in-house built, digital control system monitors the various flows and controls an immersion pump (Comet immersion pump, OCEAN, Germany) using flow-feedback from the ultrasonic flow sensor. A pressure sensor (40PC series, Honeywell Inc., Freeport, Illinois) keeps the pump operating in the same pressure range.

## Methods

In dynamic SPECT-MPI, the distribution of an injected radiotracer bolus is recorded over time and displayed in so-called time activity curves (TACs), including the arterial input function (AIF) and tissue response curves (TRCs). Blood flow models use this information to estimate tissue perfusion levels. At this stage of phantom development and evaluation, we explored the effect of varying cardiac output (CO; L/min), myocardial flow rate (*Q*_myo_; mL/min), and injected activity (*A*_inj_; MBq) on the resulting AIF, TRC, and computed MBF.

### Phantom measurements

Seven flow measurements were executed in two measurement sessions. Pump flow (i.e. CO) was set at 1.5 and 3.0 L/min and the planned activity administration between 350 and 550 MBq to evaluate their effect on AIF measurement. Reproducibility of the AIF curve was measured by fivefold repetition (CO = 1.5 L/min, *A*_inj_ ≈ 350 MBq). These five measurements were also used to evaluate TRC simulation in the three myocardial segments, including reproducibility measurements. This resulted in 15 TRCs in total. Each myocardial segment contained a different inlay to investigate which one was most suitable for the measurement of tissue perfusion. The three myocardial segments comprised:

- a basic one-compartment with no inlay (basic 1C),
- a one-compartment with sponge inlay (sponge 1C) and
- a two-compartment created by three perforated tubes (tubes 2C) (see Fig. 1(A)).

In the measurements, average flow measured in the myocardial segments (*Q*_myo_) served as reference standard and was varied between 50 and 150 mL/min.

### Myocardial perfusion imaging

All data were acquired in list mode using a cadmium-zinc-telluride SPECT system (D-SPECT, Spectrum Dynamics, Caesarea, Israel). The standard clinical protocol consisted of 6 min dynamic scanning, starting just before injection of a radiotracer bolus. Two millilitres of 330–550 MBq ^99m^Tc-tetrofosmin solution was injected at 1 mL/s, followed by a 20-mL saline flush. Typically, data was re-binned into 32 frames consisting of 21 frames of 3 s; 4 frames of respectively 9, 15, 21 and 27 s; and 7 frames of 30 s. An OSEM technique was used for reconstruction of dynamic imaging acquisitions, with 4 iterations and 32 subsets [19].

### Data-analysis

**Fig. 3:**
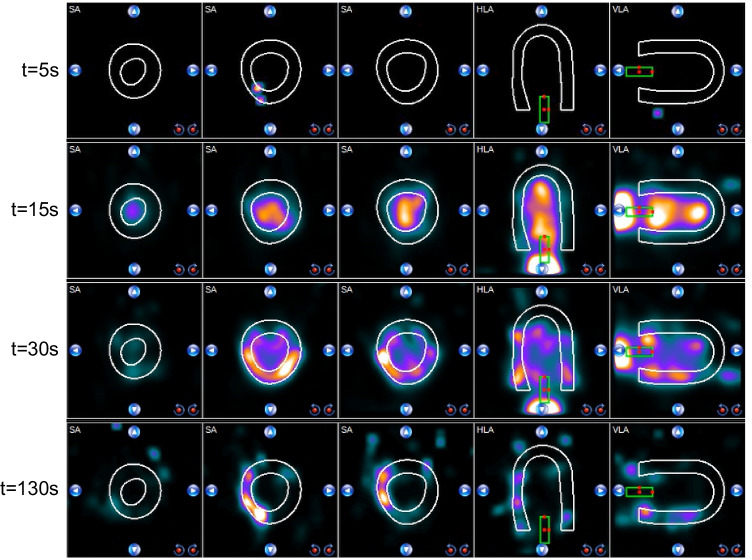
Time lapse of radiotracer distribution in the myocardial perfusion phantom visualised by clinical analysis software. The white contours indicate the myocardial tissue region of interest (ROI) and the green box the arterial ROI. The visualised coronary territories correspond to different myocardial tissue inlays in the phantom. SA = short axis, HLA = horizontal long axis, VLA = vertical long axis

Resulting dynamic image datasets were analysed with clinical software (Corridor4DM software, INVIA Medical Imaging Solutions, United States). In this software, myocardial surfaces were algorithmically estimated from summed myocardial images [19], indicating the region of interest (ROI) for TRC measurement. The AIF was derived from a manually selected ROI in the LVC using a default size box placed at the centre of the LVC (see Fig. 3). The software displayed the AIF, TRCs and computed MBF for different anatomical regions. We selected the display of the three main coronary regions to match the visualised data with the myocardial segments of the phantom.

The blood flow model applied in the clinical MBF analysis software was a net retention model proposed by Leppo et al. [20] and Yoshida et al. [21]. The following modified equation was used to calculate the retention rate (R) of tracer in the myocardium in millilitres/gram/minute at time *t*. This myocardial uptake can be expressed as the product of the MBF (mL/g/min) and the extraction fraction (*E*):

$$R=\mathrm{MBF}\times E=\frac{\frac{1}{PV({t}_{3}-{t}_{2})}{\int }_{{t}_{2}}^{{t}_{3}}\left(P\left(t\right)-{S}_{m}{C}_{a}(t)\right)dt}{CF{\int }_{0}^{{t}_{1}}{(C}_{a}\left(t\right)-{S}_{b}P(t))dt}$$

In this, *C*_a_(*t*) and *P*(*t*) correspond to the average arterial and tissue tracer concentration over time, the AIF and TRC respectively. Integration limit *t*_1_ denotes the end of the blood pool phase (typically at 1.5 min), whereas *t*_2_ and *t*_3_ denote integration limits of the average tissue activity (typically from 1.5 to 2.5 min). In our measurements, *t*_2_ and *t*_3_ were set to frames 15 and 20 (around 40‒60 s), due to the absence of tracer trapping and recirculation in the perfusion phantom model. Several corrections were applied to the data to compute absolute flow. Firstly, the acquired myocardial counts were corrected for partial volume losses using a recovery coefficient for the myocardium (PV). Partial volume effects also occurred in the measurement of blood-pool activity, whereby subsequent decrease in AIF was compensated by a cross-calibration factor (CF). Finally, theoretically computed spillover fractions, *S*_m_ and *S*_b_, correct the spillover from the blood pool activity to the myocardium, and vice versa [15, 16, 22].

TACs of all phantom measurements were exported to Matlab (2016a; The MathWorks Inc, Natick, Mass) for further data analysis, visualisation and comparison with a patient example (adapted from [23]). Reproducibility of phantom-based TAC measurement was evaluated by comparing the obtained area under the curve (AUC). The mean AUC and standard deviation were calculated for all 5 AIFs and 9 (3 × 3) TRCs. In this, the injected radiotracer activity was first normalised to 350 MBq. The relation between *Q*_myo_ and computed MBF was described with Pearson correlation statistics.

## Results

### Dynamic perfusion images

Figure 3 shows a typical perfusion image time lapse of the radiotracer distribution in the myocardial perfusion phantom along the short, horizontal and vertical long axes. At *t* = 15 s, passing of the diluted radiotracer bolus is captured in the LVC. Subsequently, a part of the radiotracer solution flows into the myocardial segments, which is shown at *t* = 30 s and *t* = 130 s. The matching dynamic perfusion imaging video can be found in the Supplement.

### Time activity curve analysis

**Fig. 4:**
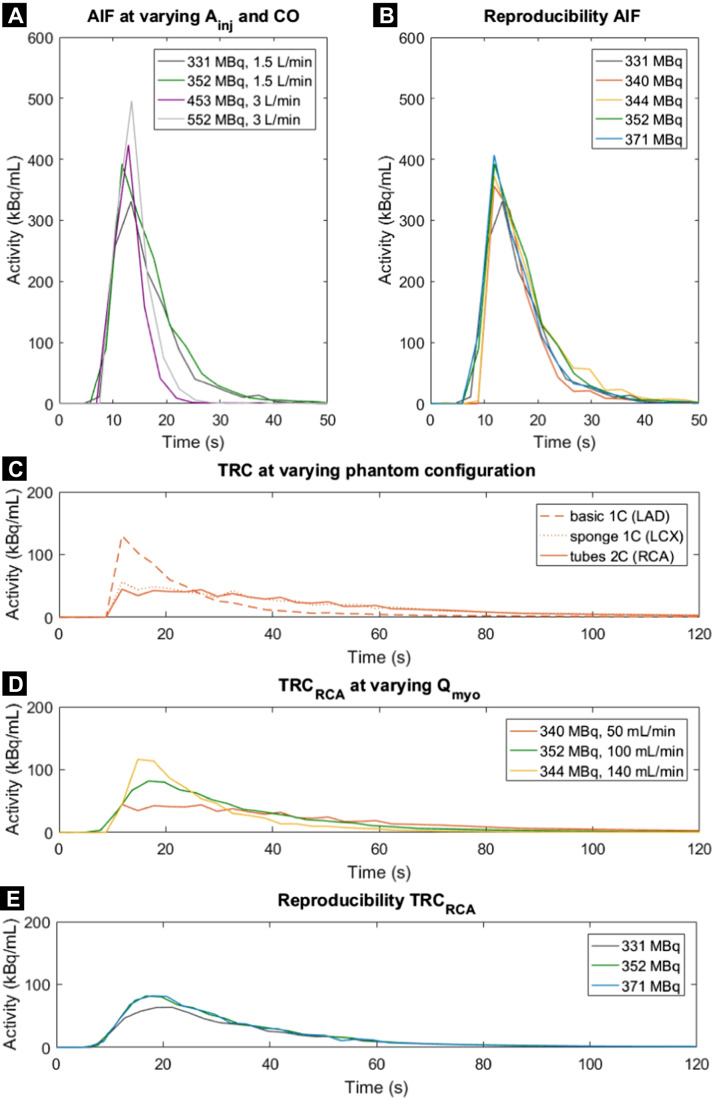
**A**–**E** Time activity curves obtained using the myocardial perfusion phantom. Arterial input functions (AIFs) were acquired in the left ventricle at varying injected activity of ^99m^Tc-tetrofosmin (*A*_inj_) and cardiac output (CO). Resulting tissue response curves (TRCs) in the three myocardial segments were executed at varying myocardial flow rates (*Q*_myo_) and tissue inlays (1 or 2 compartments). Each line colour denotes a single flow measurement (*n* = 7). LAD = left anterior descending coronary artery, RCA = right coronary artery, LCX = left circumflex coronary artery

**Fig. 5:**
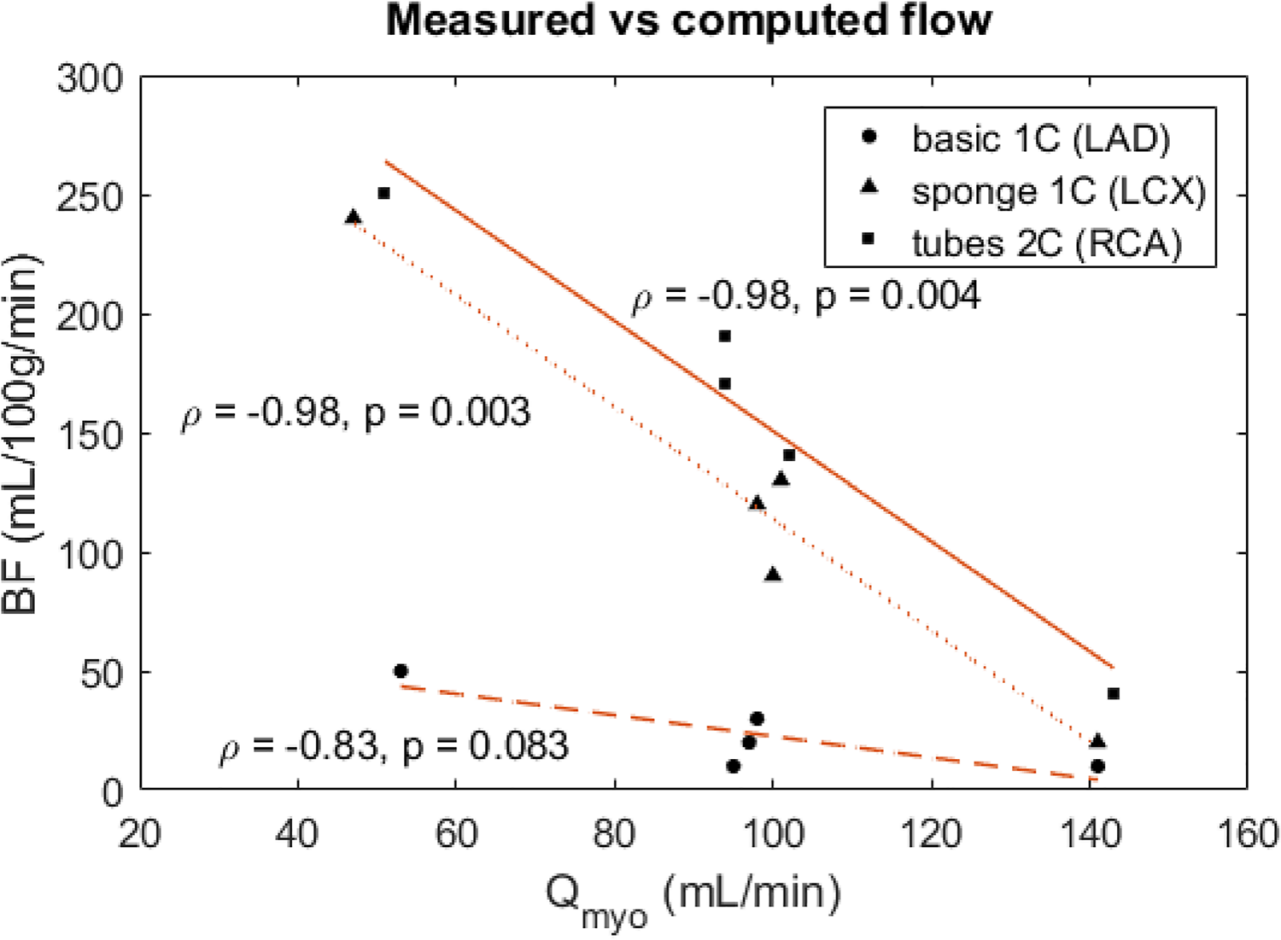
Correlation plot of reference flow (*Q*_myo_) and computed myocardial blood flow (MBF) for three different phantom tissue inlays (1 or 2 compartment). $\rho$ = Pearson correlation coefficient, LAD = left anterior descending coronary artery, RCA = right coronary artery, LCX = left circumflex coronary artery

Resulting TACs of the AIF and TRC are displayed for varying settings (Fig. 4). As can be seen, injection of larger radiotracer bolus activity corresponds to higher observed peak activity. Moreover, at an increased CO (i.e. set pump flow), the AIF exhibits a shorter retention time. Repeated measurements show good AIF reproducibility (Fig. 4B and Table 1). Measured TRCs generally show a higher peak activity for the basic 1C configuration, though the retention time of the radiotracer is longer for the sponge 1C and tubes 2C (Fig. 4C). An increased *Q*_myo_ generally results in higher measured peak activity and shorter retention time. TRC measurements demonstrate good reproducibility as well (Fig. 4E and Table 1). Figure 5 shows the correlation between measured and computed flow in the three myocardial segments, whereby each segment corresponds to a different tissue inlay. Pearson correlation coefficient $\rho$ is largest for measurements with the sponge 1C and tubes 2C ($\rho$ = − 0.98).

**Table 1:** Reproducibility of phantom based time activity curve measurement. The mean and standard deviation (SD) were calculated for the areas under the curve (AUCs) of both the arterial input function (AIF) and tissue response curves (TRCs) for *n* measurements. The TRCs correspond with the three coronary regions that have a varying tissue inlay (1 or 2 compartments)

	AUC (MBq mL^−1^ s)	
	Mean (SD)	*n*
Normalised AIF	3.77 (0.33)	5
Normalised TRC:		
basic 1C (LAD)	1.34 (0.12)	3
sponge 1C (LCX)	2.40 (0.08)	3
tubes 2C (RCA)	2.33 (0.11)	3

*LAD* left anterior descending coronary artery, *LCX* left circumflex coronary artery, *RCA* right coronary artery

### Patient data comparison

**Fig. 6:**
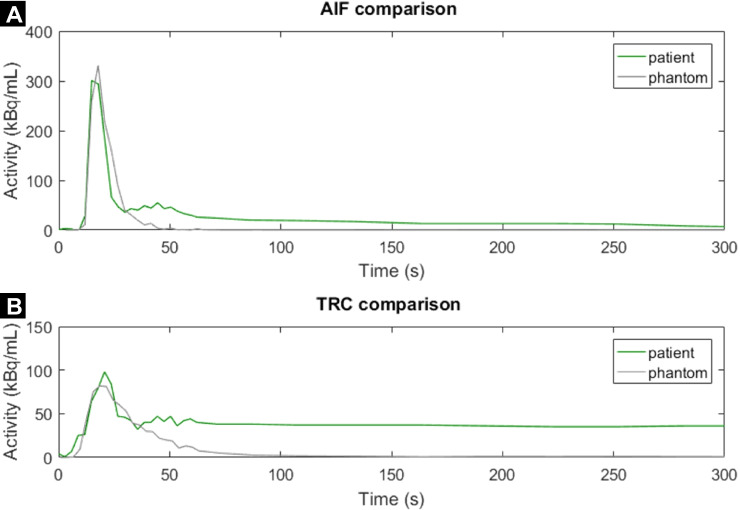
**A**, **B** Time activity curves comparing normal patient data with phantom data. The patient example is adapted from [23]. AIF = arterial input function, TRC = tissue response curve

**Fig. 7:**
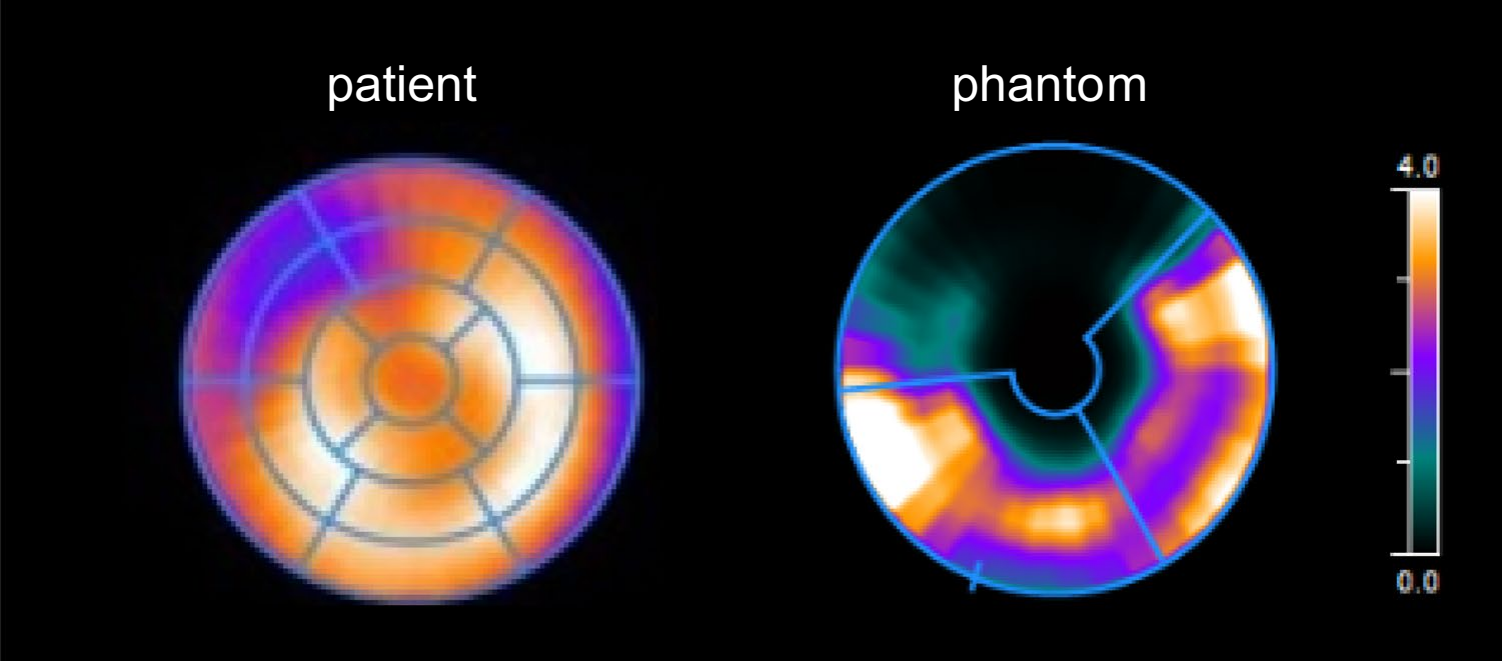
Myocardial blood flow (MBF) bull’s eye plot comparison between example patient and phantom data. Both plots indicate a perfusion deficit in the coronary territory supplied by the left anterior descending coronary artery. The patient example is adapted from [23]. In the phantom, apex simulation was disregarded. AIF = arterial input function, TRC = tissue response curve

Figures 6 and 7 match phantom data with patient data. The phantom-based AIF closely resembles the shape of the AIF in a patient (Fig. 6A). The phantom-based TRC is comparable to that of a patient, though it has a substantially shorter retention time. Figure 7 shows an example of a MBF analysis in a patient and a phantom, both indicating a perfusion deficit in the LAD region.

## Discussion

To our knowledge, this paper presents the first myocardial perfusion phantom described in literature, dedicated to quantitative evaluation of clinical MPI hard- *and* software. We have successfully measured first-pass LV flow and myocardial perfusion in SPECT-MPI. In addition, we have performed initial reproducibility measurements and initial sensitivity analyses on TAC measurement and MBF computation for the following parameters: CO, *Q*_myo_ and *A*_inj_.

### Phantom design, realisation and testing

The phantom resembles the anatomy and (patho-) physiology of LV flow and myocardial perfusion in many respects. Nonetheless, several assumptions and simplifications have been applied in its design and realisation. First, we focused on measuring first-pass myocardial perfusion; hence, tracer recirculation and uptake in surrounding tissue were disregarded. Second, we designed a stationary perfusion phantom constructed of rigid plastic, which does not model cardiac contraction, nor has a physiological flow profile. Third, we also excluded simulation of the apex. Last, the current inlays of the myocardial segments only mimic tissue perfusion as a semi-controllable two-compartment representation (i.e. no tracer trapping).

We have carried out multiple measurements to investigate how accurately and reproducibly the phantom resembles myocardial perfusion, given the simplifications that were made. Accordingly, the acquired phantom TAC curves are like those in patients in multiple ways, e.g. the shape of the curves and their activity range. Aside from these promising results, we also noticed some dissimilarities. For example, we observe an additional peak in the patient-based AIF around *t* = 45 s (Fig. 6A). This peak can be explained by (unwanted) spillover activity from the myocardium to the blood pool. By enlarging and narrowing the arterial ROI (green box in Fig. 3), it becomes possible to create and control similar spillover effects in our phantom setup. Secondly, we observed shorter retention times for the phantom TRCs compared to those obtained in patients (Fig. 6B). The missing tracer trapping, and recirculation can explain this effect.

In the phantom TAC analysis, the obtained TRCs showed a higher peak activity and shorter retention time at increasing *Q*_myo_, which agrees with our expectations and enables the opportunity to measure local and global perfusion deficits. In addition, the phantom comprised of three different myocardial segment inlays to enable investigation of which one is most suitable for measuring tissue perfusion. The basic 1C inlay yielded the shortest retention time and showed an uneven distribution of tracer within the compartment. The other two inlays, i.e. the sponge 1C and tubes 2C, presented more favourable tracer dynamic behaviour, though distribution of tracer within the segment can be further optimised (see Fig. 4). We expected that a two-compartment inlay ensures an even longer retention time, but due to the low number of perforated tubes, this effect became insignificant. The underlying cause of the observed uneven tracer distribution seems to be jet formation at the three entrance points of each myocardial segment. This might be an oversimplification in mimicking the physiological precapillary network. Its effect is most present in the absence of simulated tissue or capillaries.

Figure 5 visualises the possibility of comparing set *Q*_myo_ in the phantom setup with computed BF derived from clinical hard- and software. The observed high, linear correlation is physiologically incorrect (at this stage of phantom development) and is comprised of a relatively small dataset. Nevertheless, in this proof-of-concept stage, these measurements confirm what is being simulated and give us a glimpse of future phantom potential. In addition, Fig. 7 exemplifies the possibility to simulate perfusion deficits. The underlying cause of the severe perfusion deficit mimicked in the LAD region is (1) the use of a basic 1C tissue inlay (shortest retention time of tracer) and (2) a low *Q*_myo_ of 50 mL/min. These settings resulted in a computed BF of around 50 mL/100 g/min in the LAD region (see Fig. 5).

The obtained results are comparable to the performance of other perfusion phantoms. Li et al. [24] compared tissue perfusion with a dialysis cartridge to a basic spherical-shaped volume using US imaging. Kim et al. [25] performed a similar analysis in MRI by comparing a dialysis cartridge with a gel bead–filled perfusion phantom. Both studies showed an even distribution of contrast agent with a prolonged retention time using the numerous permeable fibres of the dialysis cartridge. Such dialysis cartridges are therefore frequently used to model tissue perfusion [16]. Nonetheless, the tracer uptake in myocardial tissue is uncontrolled in these phantom models and simulation of organ anatomy (e.g. heart contours) is being disregarded. In our experimental setup, we observe a promising correlation between reference flow and computed MBF, which is comparable to other perfusion phantom studies [16].

### Phantom validation and application

Justification of the choices made in phantom design and testing depends on the intended phantom application. In our design, most notable choices are the absence of cardiac contraction, tracer trapping and recirculation, together with the relatively low CO that was applied in our measurements. These choices and simplifications are considered acceptable if we are predominantly interested in the evaluation of MPI system responses. In such evaluations, excellent reproducibility of phantom performance is most essential. A more advanced myocardial perfusion phantom may broaden its application domain; however, an increase in complexity is difficult to achieve in terms of controllability and phantom validation. Our initial TAC measurements show good reproducibility, as shown in Fig. 4 and displayed in Table 1. Table 1 demonstrates that the AUCs from the obtained AIFs and TRCs were quite similar for repeated measurements (SD is below 10% of mean AUC), which can be even further optimised in future work (e.g. by removing the presence of unwanted air bubbles).

In this study, our phantom application domain was restricted to SPECT-MPI. However, the phantom could be applied in other imaging modalities as well. At this moment, phantom-based evaluation of dynamic SPECT-MPI is of major relevance, since recent advances in detector-technology speculates it may become technically feasible to perform quantitative MBF and MFR analysis with SPECT-MPI [26]. We are confident that perfusion phantom studies will make a valuable contribution to this research domain.

Overall, we present a novel myocardial perfusion phantom with add-on myocardial segments that has the possibility to mimic regional and global perfusion deficits and is unique in its compatibility with clinical hard- and software. This combination of features can be of interest in the validation of blood flow models, for example, to evaluate effects of attenuation and spillover on TAC measurement and MBF computation. Other foreseen applications involve intra- and intermodal comparison. Further phantom testing and validation is needed to achieve these goals.

## Conclusions

This proof-of-concept paper demonstrates we have successfully measured first-pass LV flow and myocardial perfusion in SPECT-MPI using a novel, dedicated, myocardial perfusion phantom. The resulting AIFs and TRCs show good reproducibility and resemble a patient TAC curve on multiple aspects. Other phantom requirements were also met, including the acceptance of phantom contours by commercial MBF analysis software and the simulation of local and global perfusion deficits. Moreover, the phantom’s modular and 3D printed design enables rapid prototyping, allowing us to further improve on the measurement and evaluation of radiotracer/contrast kinetics in dynamic, multimodal MPI applications.

## Supplementary Information

Below is the link to the electronic supplementary material.

Supplementary file1 (MP4 3145 KB)
