# Correction to: Development of a dedicated 3D printed myocardial perfusion phantom: proof-of-concept in dynamic SPECT

**DOI:** 10.1007/s11517-022-02512-4

**Published:** 2022-04-08

**Authors:** Marije E. Kamphuis, Gijs J. de Vries, Henny Kuipers, Marloes Saaltink, Jacqueline Verschoor, Marcel J. W. Greuter, Riemer H. J. A. Slart, Cornelis H. Slump

**Affiliations:** 1 Multi-Modality Medical Imaging (M3i) Group, Faculty of Science and Technology, Technical Medical Centre 2386, University of Twente, P.O. Box 217, 7500 AE, Enschede, The Netherlands; 2 Robotics and Mechatronics (RaM) Group, Faculty of Electrical Engineering Mathematics and Computer Science, Technical Medical Centre, University of Twente, Enschede, The Netherlands; 3 Department of Nuclear Medicine, Ziekenhuis Groep Twente, Hengelo, The Netherlands; 4 Medical Imaging Centre, Department of Nuclear Medicine and Molecular Imaging, University Medical Center Groningen, University of Groningen, Groningen, The Netherlands; 5 Biomedical Photonic Imaging Group, Faculty of Science and Technology, Technical Medical Centre, University of Twente, Enschede, The Netherlands

**Correction to: Medical & Biological Engineering & Computing**

10.1007/s11517-021-02490-z

The original article contained a mistake.

Figure 4 is not displayed correctly in the published paper. The correct Figure 4 is shown below.

In addition, the caption was Fig. 4A-D and should have been Fig. 4A-E. The correct Figure caption is included.

**Fig. 4:**
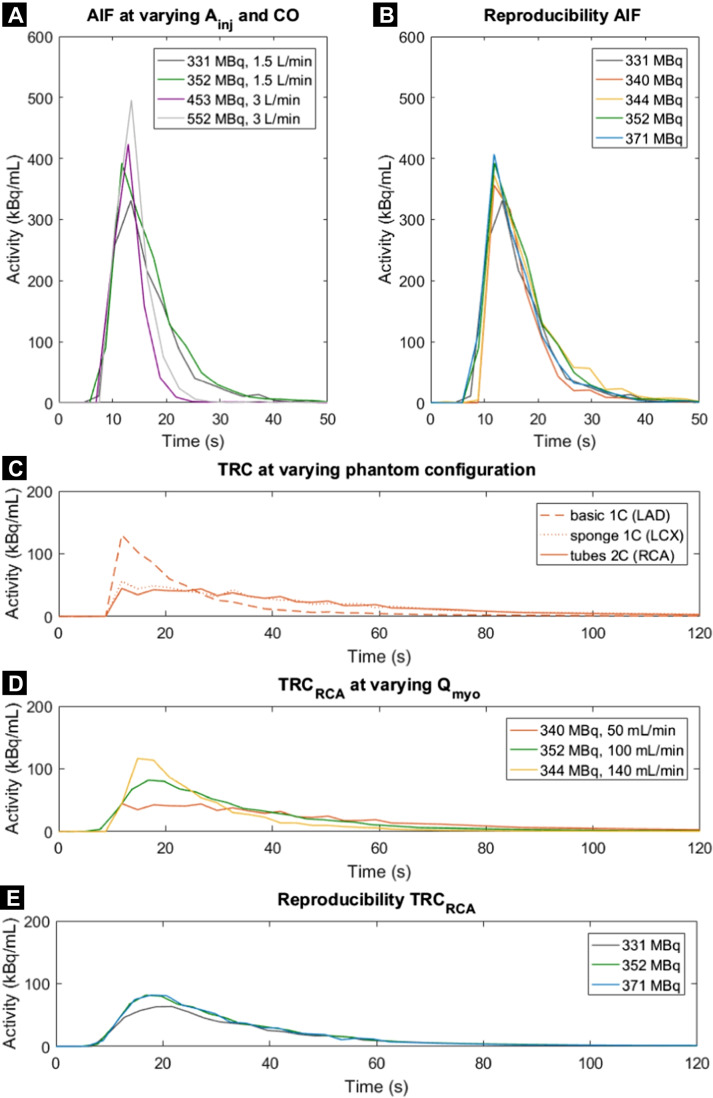
**A**–**E** Time activity curves obtained using the myocardial perfusion phantom. Arterial input functions (AIFs) were acquired in the left ventricle at varying injected activity of ^99m^Tc-tetrofosmin (A_inj_)and cardiac output (CO). Resulting tissue response curves (TRCs) in the three myocardial segments were executed at varying myocardial fow rates (Q_myo_) and tissue inlays (1 or 2 compartments). Each line colour denotes a single fow measurement (n=7). LAD=left anterior descending coronary artery, RCA=right coronary artery, LCX=left circumfex coronary artery

The original article has been corrected.

